# A single point-mutation within the melanophilin gene causes the *lavender *plumage colour dilution phenotype in the chicken

**DOI:** 10.1186/1471-2156-9-7

**Published:** 2008-01-15

**Authors:** Mohsen Vaez, Sarah A Follett, Bertrand Bed'hom, David Gourichon, Michèle Tixier-Boichard, Terry Burke

**Affiliations:** 1 Department of Animal and Plant Sciences, University of Sheffield, Sheffield S10 2TN, UK; 2 INRA, AgroParisTech, UMR1236 Génétique et Diversité Animales, F-78350, Jouy-en-Josas, Cedex, France

## Abstract

**Background:**

The *lavender *phenotype in the chicken causes the dilution of both black (eumelanin) and red/brown (phaeomelanin) pigments. Defects in three genes involved in intracellular melanosomal transport, previously described in mammals, give rise to similar diluted pigmentation phenotypes as those seen in *lavender *chickens.

**Results:**

We have used a candidate-gene approach based on an expectation of homology with mammals to isolate a gene involved in pigmentation in chicken. Comparative sequence analysis of candidate genes in the chicken identified a strong association between a mutation in the *MLPH *gene and the diluted pigmentation phenotype. This mutation results in the amino acid change R35W, at a site also associated with similar phenotypes in mice, humans and cats.

**Conclusion:**

This is the first time that an avian species with a mutation in the *MLPH *gene has been reported.

## Background

**Figure 1:**
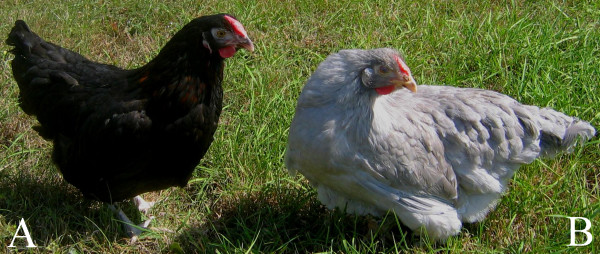
**The lavender phenotype**. Chickens expressing (a) wild type (LAV*N/LAV*N), and (b) lavender (LAV*L/LAV*L), phenotypes on an *extended black *(E*E) background.

*Lavender *(LAV*L) is an autosomal recessive mutation of the chicken (*Gallus gallus*) affecting the neural crest derived melanocytes [1]. It causes the dilution of both eumelanin and phaeomelanin to a light grey or buff, respectively (Figure 1). Light and electron microscope studies have revealed that, although *lavender *melanocytes possess relatively normal dendrite morphology, there is defective peripheral accumulation of melanosomes to the dendrites [2]. This results in the patchy transfer of melanosomes into the keratinocytes of the growing feather. The dilution effect is essentially the result of a mixture of pigmented and unpigmented regions within the feather barbs. Although *lavender *melanocytes are defective in melanosomal translocation, they show no apparent ultrastructural defect in the microfilament system [2].

Similar dilution effects can be seen in the mouse mutants *dilute*, *ashen *and *leaden*. The melanocytes of these mutants each display a similarly defective melanosomal transport system to that seen in *lavender *chickens, resulting in the patchy transfer of pigment to the keratinocytes of the hair. *Dilute*, *ashen *and *leaden *have been extensively studied and the process of peripheral accumulation of melanosomes has been shown to be controlled by an unconventional myosin, *MYOVa *[3], the ras-related GTPase *RAB27a *[4] and the Rab effector melanophilin (*MLPH*, also known as *Slac-2*) [5]. In humans, mutations in these genes have been identified in patients with the rare autosomal recessive disorder, *Griscelli syndrome *(*GS*). Three forms of *GS *have been described and, although all show pigmentary dilution of the skin and hair, additional defects have also been observed. Patients with *GS1 *carry mutations within *MYOVA *and have a severe primary neurological impairment [6,7] and some of the alleles of *MYOVa *in the mouse also show neurological defects [8]. Mutations in *RAB27A *are associated with *GS2*, and are characterised by an immune defect [9-11]. In *ashen *mice additional defects include prolonged bleeding times due to defects in the platelet-dense granules [4]. Mutations in *MLPH*, in both humans with Griscelli syndrome type III and *leaden *mice, have been shown to be associated only with hypopigmentation and no other physiological problems [12].

The products of these three genes work together to anchor melanosomes to the actin cytoskeleton, thus facilitating their transport within the cell. They form a protein complex that has been shown to be essential for the capture and movement of melanosomes via the actin cytoskeleton [13-16]. MyoVa is an actin-based motor within the melanocyte. Mlph binds with MyoVa at one end and Rab27a, which is itself targeted to the melanosomal membrane, at the other (for a full review see reference [17]).

The *MLPH *gene is well conserved between mouse and human. The protein consists of several well defined domains. At the N-terminus there are two Slp homology domains (SHD), as well as a zinc finger domain, all of which have been shown to bind Rab proteins. Binding of Myosin Va occurs towards the centre and at the N-terminus. In the human, a C103T transition was identified in an SHD in exon 1, resulting in an R35W substitution [12]. In *leaden *mice, R35 is one of seven deleted residues, REEERLQ, resulting from a 21-bp deletion [5]. In both humans and mice it is postulated that the dilution phenotype is caused by the inability of Mlph to bind Rab27a and link it to MyoVa. Brumbaugh *et al*. suggested that *leaden *in mice and *lavender *in chicken might be homologous [1], although to our knowledge this hypothesis has not been tested.

Pigment-dilution phenotypes associated with mutations in *MLPH *have also been described in several other mammals, such as cats, dogs and mink [18-22]. Experiments in Japanese quail *Coturnix coturnix japonica *using chicken-quail hybrids have indicated that the same gene is responsible for both the quail *lavender*, also known as *bleu*, and the chicken *lavender *[23]. In other bird species, similar feather colour dilutions have been reported, including the *recessive slate *turkey (*Meleagris gallopavo*) [24], *milky *pigeon (*Columba livia*) [25], and the *lavender *muscovy duck (*Cairina moschata*) [26,27]. It is as yet unknown which genes are responsible for these dilution mutations in any bird species.

Given the phenotype of *lavender *melanocytes we suspected that the melanophilin gene might underlie the *LAV *locus. Here, we present strong evidence that *lavender *in the chicken is caused by a single nucleotide substitution in the *MLPH *gene.

## Results

### Identification and expression of the avian melanophilin gene

**Figure 2:**
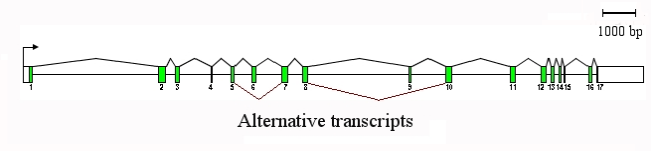
**Schematic diagram depicting the intron/exon organisation of the chicken *MLPH *gene**. Coding regions are shown shaded and UTR sequences in white. Exons 6 and/or 9 are removed by splicing in some isoforms.

**Figure 3:**
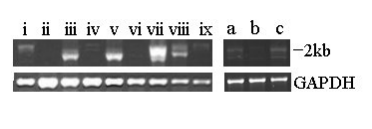
**Expression of *MLPH *in adult and embryonic tissues of the male wild-type chicken**. Tissues: (i) liver, (ii) muscle, (iii) kidney, (iv) heart, (v) lung, (vi) testis, (vii) skin, (viii) brain and (ix) spleen, and skin at ages (a) E14, (b) E17 and (c) 10 weeks. Size is indicated on the right.

*MLPH *is located on chicken chromosome 7. The genomic organisation of melanophilin is shown (Figure 2). Sequence analysis carried out on cDNA, and confirmed on genomic DNA, showed that *MLPH *consists of 17 exons that contain 2052 bp of coding sequence, translating into 683 amino acids. We found that exons 6 and/or 9 are absent in some transcripts, most probably due to alternative splicing events (Figure 3). Exon 1 of chicken *MLPH *is also homologous to exons 1 and 2 in mammals [5,12,19].

In a similar manner to the expression seen in mice [5], chicken *MLPH *is expressed predominantly in epithelial-rich tissues (Figure 3). We found no differences in the level of expression with respect to sex or genotype, except in the ovary, where expression was higher in *lavender *females (data not shown). Expression of the ubiquitously expressed *GAPDH *was constant across all samples. With respect to age, we detected the highest degree of embryonic expression at embryonic day 14 (E14), consistent with this period having the highest rate of development of feather follicles [28], reducing at E17 and then increasing again at 10 weeks, when the adult plumage is being produced (Figure 3).

### Characterisation of the causal mutation of lavender in the chicken

**Figure 4:**
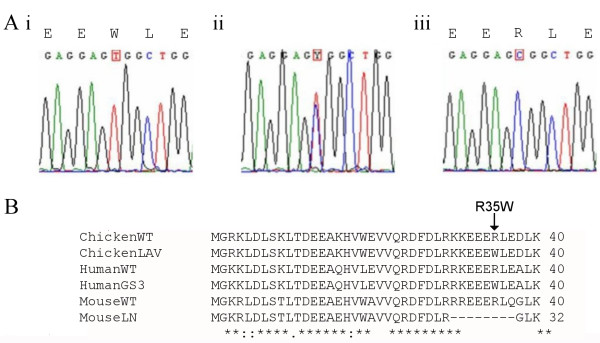
**A single point-mutation is associated with the lavender allele**. (A) Sequence traces for the part of chicken *MLPH *exon 1 showing the C103T SNP; (i)LAV*L/LAV*L, (ii) LAV*N/LAV*L, (iii) LAV*N/LAV*N. Reading frame is +1, and respective amino acids are shown above the first base pair in a codon for the homozygous sequences. (B) ClustalW alignment of the wild type (WT) and mutated (LN, GS3 and LAV, respectively) amino acid sequences from mouse [6], human [13], and chicken.

We identified 7 SNPs – 5 synonymous and 2 non-synonymous variants (C103T, non-synonymous (R35W); T996C, synonymous; A1056G, synonymous; C1284T, synonymous; A1356G, non-synonymous (I452M); G1701A, synonymous; C1824T, synonymous) – amongst the 94 chicken samples sequenced in their entirety for this study. Of the two non-synonymous SNPs (Table 1), only one was found to be associated with the *lavender *phenotype (Figure 4A). All the other SNPs were found to be polymorphic in wild type birds. This single point-mutation, found in exon 1, 103 bp from the start codon, results in the amino acid change R35W (where an Argenine in the wild type is replaced by a Tryptophan), which is the same mutation reported in humans with Griscelli syndrome type III [12], and is also included in the deletion found in *leaden *mice [5] (Figure 4B).

**Table 1:** Two non-synonomous SNPs in the coding sequence of *MLPH *in a selection of different chicken phenotypes.

**Phenotype**	**Nucleotide at position 103**			**Nucleotide at position 1356**		
	
	**C/C**	**C/T**	**T/T**	**A/A**	**A/G**	**G/G**
White	27	0	0	18	5	4
Silver	7	0	0	7	0	0
Black/Brown	13	14	0	24	0	3
Mottled	18	0	0	18	0	0
Lavender	0	0	15	0	0	15

Only base pair position 103, causing the R35W substitution, was shared by all the *lavender *phenotypes studied and those that were expected to be heterozygous at this locus.

**Figure 5:**
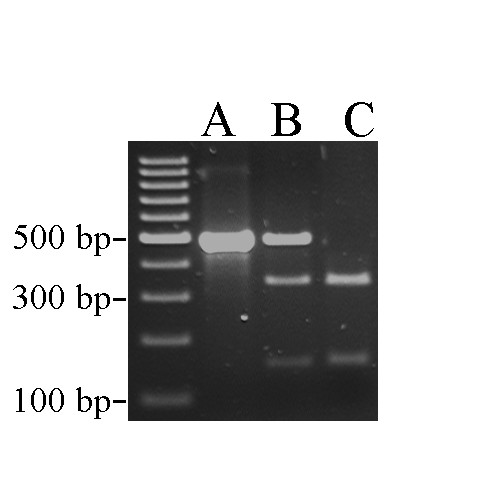
PCR-RFLP analysis for the presence/absence of the LAV*L allele. PCR amplification from genomic DNA results in a band of size 480 bp. Digestion with *Bsr*BI cuts the wild type LAV*N allele into two fragments of sizes 334 bp and 146 bp, while the LAV*L allele does not digest; (A) LAV*L/LAV*L, (B) LAV*N/LAV*L, (C) LAV*N/LAV*N.

The C103T mutation results in the loss of the single restriction site for the *Bsr*BI restriction enzyme, which cuts the sequence CCGCTC, found only in the wild-type LAV*N allele. This meant that we could design a simple RFLP test in order to identify the presence or absence of the *lavender *allele. Using a PCR product amplified from genomic DNA, the LAV*N allele is digested into two fragments of sizes 334 bp and 146 bp by *Bsr*BI, while the LAV*L allele remains intact at 480 bp (Figure 5).

We obtained the RFLP genotype for chickens of known *lavender *genotype in the Nouzilly pedigrees. For the 159 individuals tested in this manner, we found complete association between the *lavender *allele and the presence of the C103T SNP: all 46 individuals with the *lavender *phenotype were homozygous for the 480-bp allele and all birds known to be heterozygous for LAV*N/LAV*L (offspring of four *lavender *dams mated with a wild-type sire) were heterozygous at C103T.

**Figure 6:**
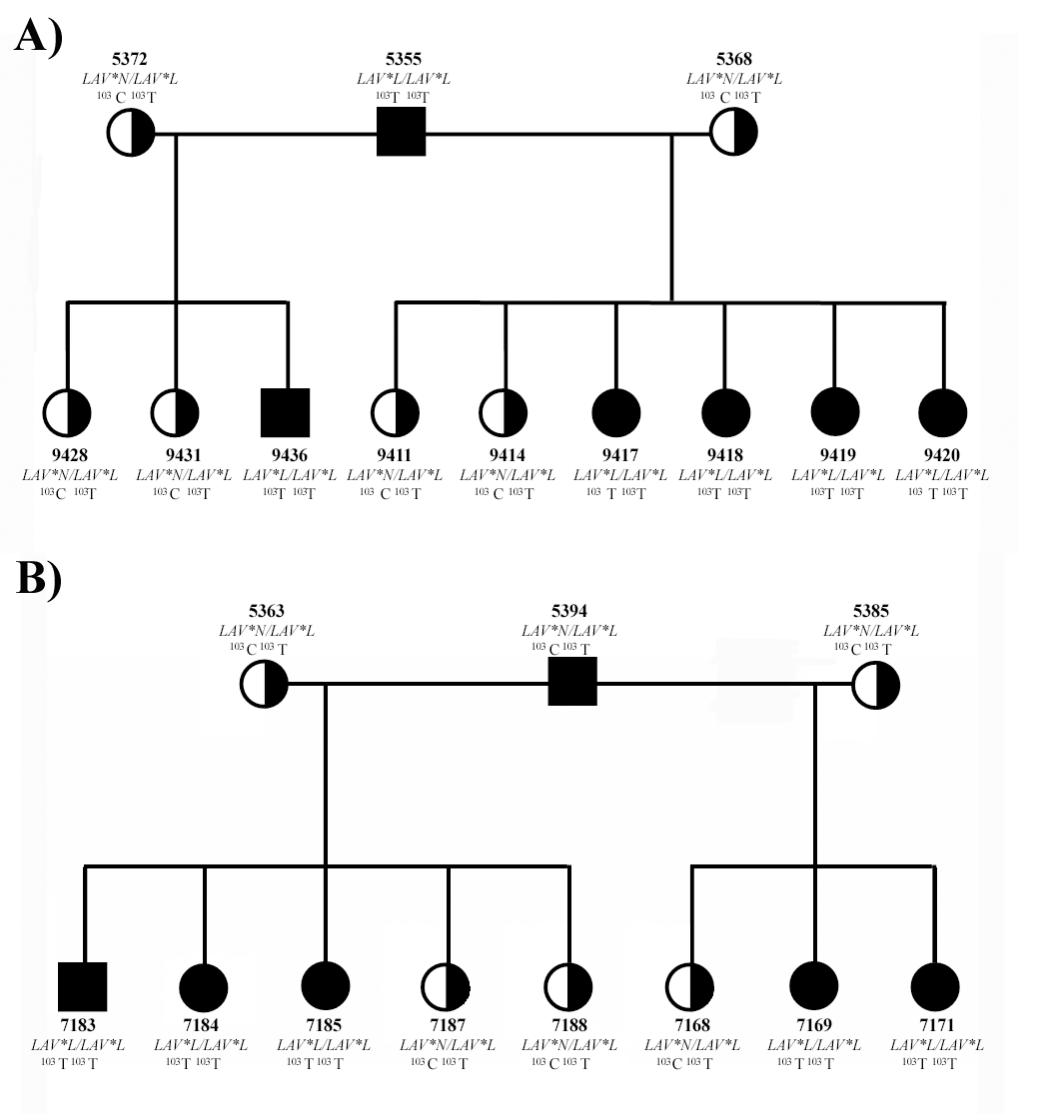
**Pedigree of a lavender allele family**. Segregation of the putative ^103^T *lavender *allele in crosses between two homozygous *lavender *sires, A) 5355 and B) 5394, and four heterozygous dams, A) 5368, 5372 and B) 5363, 5385, is consistent with the *lavender *phenotype. Males are denoted by squares, and females by circles. *Lavender *(LAV*L) and wild-type (LAV*N) haplotypes are indicated as solid black and solid white symbols, respectively.

Analysis of cosegregation in a pedigree confirmed this result (Figure 6). Several informative families were produced by mating heterozygous dams to homozygous lavender sires (Figure 6). The segregation of haplotypes was completely in accordance with the observed phenotypes (LOD = 3.91).

## Discussion

We have used a comparative-gene approach to identify the mutation underlying the *lavender *phenotype in the chicken. *Lavender *dilutes plumage colour in a manner comparable to the diluted coat phenotypes of the mouse mutants *dilute*, *ashen *and *leaden *[3-5]. Comparisons at the ultrastructural level, between *lavender *and *leaden *melanocytes, together with the physiological similarities of both mutants – showing no harmful effects in each case – led us to hypothesise that the same gene, melanophilin, might be responsible.

Sequence analysis of the coding sequence of *MLPH *revealed a non-synonymous substitution in exon 1 that segregated perfectly with the LAV*L allele. This SNP, C103T, results in the amino acid change R35W. The exact same mutation was also found in humans with Griscelli syndrome type III [12]. The translation of the first 120 base pairs of sequence from the start codon of the chicken *MLPH *gene (Figure 4B) shares 87% and 77% sequence identity, with E values of 1e-9 and 3e-9, respectively, with the human and mouse sequences. It has been postulated that the mutation results in a change in the 3-dimensional structure of the protein, so altering the ability of the Mlph protein to act as an effective linker between Rab27a and Myosin 5 [12]. Disruption of this triprotein complex reduces the capacity for melanosome translocation to the periphery of the cell in readiness for transfer to the keratinocytes of the developing feather. Methylation is a potent mutagen and it is known that there is a bias in GC-rich regions towards the methyl-induced mutation of CpG residues into TpG residues [29]. The C130T mutation, now found in chickens as well as humans, is found at a CpG site, which may explain why this same mutation has occurred multiple times during evolution.

The genomic structure of the chicken *MLPH *gene is very similar, though not identical, to that of the mouse, human, dog and cat genes. Differences were observed with respect to the chicken exon 9, which is lacking from all the mammalian species. Exon 4, which is similar to exon 5 in the dog gene, is also absent in the human, mouse and cat sequences. In addition, exon 8 (exon 9 in human and mouse) has not been identified in dog and cat. The promoter and start codon of chicken *MLPH *are located in exon 1, while in humans the start codon is located in exon 2 [12].

The splicing events that we observed in chicken have also been reported in mammals. Our data confirm that there are no splicing differences between the *lavender *and wild-type alleles, and the same has been reported in humans. However, a recent study in the dog suggests that a SNP at the end of the untranslated exon 1 causes a slicing defect which results in reduced levels of the MLPH transcript in dogs with diluted coat colours [22]. This is a novel kind of mutation in MLPH, not previously seen before. In the chicken MLPH gene it has not yet been possible to determine the sites of alternative splicing events responsible for the different transcripts seen in both wild type and *lavender *samples. However, splicing signals can act from either close or distant positions from splice sites. Thus, it is sometimes difficult to identify the causal sites for alternative splicing events [30].

## Conclusion

A mutation in *MLPH *has occurred independently in the evolution of several domesticated animal species [18,24], often in the same, apparently highly mutable, location within exon 1. A number of other domesticated bird species also display a similar diluted phenotype, suggesting that melanophilin could also be a good candidate for these mutations. Several diluted phenotypes have been described in chickens, and molecular genetics is now starting to unravel the mechanisms underlying this diversity of plumage morphs that has been selected for during domestication.

## Methods

### *In-silico *identification of a homologue of melanophilin in the chicken

The protein sequence of the murine melanophilin gene (NM053015) was used to search for a homologous gene in the chicken genome (The Sequencing Centre, Washington University, St Louis) using TBLASTN [31]. Two contigs, accession numbers AADN01050916 and AADN01050915, demonstrated a high degree of similarity to the murine protein sequence. The contigs were analysed *in silico *in order to predict the chicken *MLPH *gene sequence using homology and gene prediction programs, primarily GENSCAN [32], to yield a sequence containing the full-length coding region of the chicken melanophilin. The predicted mRNA sequence and genomic structure were used to design primers for DNA and cDNA amplification to verify the sequence (all primer sequences are shown in Table 2). All primer pairs used in these experiments were designed using Primer3 [33]. The final mRNA sequences were verified and submitted to EMBL (EU007437-40).

**Table 2:** Primer sequences.

Name	Position	Direction	Sequence 5'-3'
ML5'-1	5'-UTR	Forward	GACTGCCCGTGGCCCCATTATTTG
ML1F	5'-UTR	Forward	GCTTTAAGACCGGTTGGATCTAC
SJ93	Exon 1	Forward	GGGGAGGAAGCTGGATCTCTC
RACE4F	Exon1	Forward	ATGGGGAGGAAGCTGGATCTCTCCAAG
RACE2R	Exon 1	Reverse	ACCTCCCAGACATGCTTGGCCTCCTC
RFLP-F1	Intron 1	Reverse	ACACTGACACAGCTGATAACATCAC
MV2R	Exon 2	Forward	GAAGTGCAAGATAGACCAGGAAAG
RACE1R	Exon 2	Reverse	GCTTGCTGTTCAGCAGGAACTTGAAGG
MV18F	Exon 3	Forward	GAAGGGGAATTCAGCTCTTCTAGG
ML14F	Exon 5–6	Forward	CAGAGTGCTACAGCAGAATGC
BlotE91F	Exon 8–9	Forward	CTCGGGAAGTCAGATTCATGAGC
ML3R	Exon 10	Reverse	CTTTCTCTTCAACTCCTCCTCCTC
ML16F	Exon 12	Forward	GTCTACCTGACTGCTGGGAAGG
RACE5F	Exon 15	Forward	GCCACAGGTCCTCAGGAGGAGGTTC
MV8R	Exon 16	Forward	ATGACTCCTTTGACCGCAACTC
MV6F	Exon 17	Reverse	AGGACAGGTGGGTCATCACTG
ML15R	Exon 17	Reverse	CTCAGGACAGGTGGGTCATCACTG
ML3'-1	3'-UTR	Reverse	GATGAGGCAGGGTGGCAGGACCAG

Only base pair position 103, causing the R35W substitution, was shared by all the *lavender *phenotypes studied and those that were expected to be heterozygous at this locus.

### Tissue samples

Four informative families were produced for pedigree analysis at the experimental facilities of the Institut National de la Recherche Agronomique (INRA), located in Nouzilly, by mating two homozygous *lavender *(LAV*L/LAV*L) sires to four heterozygous (LAV*L/LAV*N) dams. In total, ten progeny were scored for the homozygous presence of the LAV*L allele, along with seven heterozygote individuals at the *LAV *locus that displayed undiluted black plumage colour, due to their *extended black *background. These samples were used in the PCR-RFLP analysis.

A further 75 birds were also produced from the INRA flock's *lavender *line. The full MLPH coding sequence from 15 of the *lavender *birds and 14 of the heterozygotes produced from some of these breedings were sequenced in their entirety. The rest, a mixture of LAV*L/LAV*L and LAV*N/LAV*L, were analysed by PCR-RFLP only.

One hundred and fifty progeny, homozygous for the wild type LAV*N allele, were produced from several different breeds within the INRA flock, displaying a range of different, but undiluted, plumage phenotypes. 65 of these were used for sequencing, while the rest were tested for the absence of the *lavender *allele by PCR-RFLP.

Five chickens with the *lavender *phenotype were also sourced from two alternative farms in the UK and used for the PCR-RFLP analysis.

Dorsal skin tissue containing developing feather follicles was collected from embryos that had been incubated together until egg day 14 or 17, dependent on the particular breed. In the case of adult tissue samples, feathers were plucked from a patch on the dorsal surface of each bird to induce synchronised follicle development and then the birds were sacrificed 2 weeks later. All tissue samples were immediately submersed in RNAlater and stored at -20°C. In the case of adults, other tissues (liver, muscle, kidney, heart, lung, testis, spleen, brain, and ovary) were also collected at the same time and stored in a similar way to the skin samples.

### Isolation of total RNA

Up to 100 mg tissue in RNAlater (Ambion, Austin, TX, USA) was homogenised in TRIzol (Invitrogen, Paisley, UK) using a TissueLyser (Qiagen, Crawley, UK). Total RNA was isolated according to the manufacturer's protocol (Invitrogen). RNA quality and quantity were evaluated with the RNA 6000 Nano LabChip kit on the Agilent 2100 Bioanalyzer (Agilent Technologies, South Queensferry, UK) and then stored at -80°C. Subsequent DNA extractions were also carried out using the normal TRIzol protocol.

### Reverse transcription PCR and 5'-/3'-RACE

All RT-PCR reactions were performed on cDNA synthesised from 2 μg of RNA using the RETROscript kit (Ambion), according to the manufacturer's protocol, using oligo-dT in a 20-μl reaction volume. Amplification reactions followed standard PCR protocols in a volume of 50 μl, using a 1.5-μl aliquot of the RT reaction mixture, 1.5 mM MgCl_2_, 0.2 mM of each dNTP, 0.04 μM each of the primers RACE4F and ML15R (Table 2), and 1.25 units Taq DNA polymerase (Bioline). PCR was initiated by denaturation for 2 minutes at 94°C, followed by 35 cycles consisting of 30 seconds at 94°C, 1 minute at 64°C and 3 minutes at 72°C, and completed by a final extension of 5 minutes at 72°C. Twenty microlitres of the amplification product was visualised on a 1.5% agarose-TAE gel stained with ethidium bromide.

To determine the 5'- and 3'-regions, 5' and 3' RACE was performed using the SMART RACE cDNA Amplification Kit (Clontech) according to the manufacturer's instructions, with 1 μg of total RNA from chicken using the primers RACE1R, RACE2R, RACE5F and MV8R (Table 2); the products were then separated by agarose gel electrophoresis, gel purified, cloned and sequenced.

### Sequencing and subcloning

PCR products were directly sequenced, and also following cloning, on an Applied Biosystems (ABI) model 3730 DNA sequencer (ABI, Warrington, UK) and the sequencing reactions were carried out using BigDye Terminator v1.1 Cycle Sequencing kit (ABI) under the following conditions: 26 cycles of 94°C for 20 seconds, 52°C for 20 seconds and 60°C for 4 minutes. Sequencing reactions were purified and cleaned by ethanol precipitation. The primers used for initial amplification were subsequently used for sequencing with additional internal primers (Table 2) SJ93, MV2R, MV18F, ML14F, BlotE91F, ML3R, ML16F and MV6F, allowing the whole sequence to be overlapped and confirmed on both strands.

The full coding sequence of the *MLPH *transcript, including some 5'-UTR and 3'-polyA sequence, was amplified by PCR using primers ML5'-1 and ML3'-1, and was subsequently cloned into the vector pGEM-T Easy (Promega, UK) according to the manufacturer's instructions. The plasmids were completely sequenced in both directions using the universal M13 primers (forward and reverse).

The sequences obtained were edited and aligned using ClustalW software [34] and the Proseq v.2.91 software [35], then re-edited and realigned manually.

### PCR-RFLP analysis

A fragment of genomic DNA was amplified for PCR-RFLP analysis using primers ML1F, upstream of the start codon, and RFLP-F1, in intron 1 (Table 2), using standard PCR protocols in a volume of 50 μl that included 1.5 μl of genomic DNA (100 ng/μl), 1.5 mM MgCl_2_, 0.2 mM of each dNTP, 0.04 μM each of primer, and 1.25 units Taq DNA polymerase (Bioline). A standard PCR program was used of 40 cycles with a 1-minute annealing step at 58°C and a single extension step of three min at 72°C. Ten microlitres of PCR product was digested in a standard restriction digestion protocol using the *Bsr*BI restriction enzyme (BioLab). Twenty microlitres of the digestion product was visualized on a 1.5% agarose-TAE gel stained with ethidium bromide.

## Authors' contributions

MV carried out the majority of the molecular genetic studies and *in-silico *analysis and co-prepared the manuscript. SAF assisted with the molecular genetic work and drafted the manuscript. DG is in charge of the experimental farm at INRA, Nouzilly, and supervised the production of the birds and the sampling stages. BB participated in the association study by analysing informative families and unrelated breeds. MT-B participated in the design and coordination of the study and supplied the samples. TB conceived and led the study, and participated in its design and coordination and helped to draft and revise the manuscript. All authors read and approved the final manuscript.
